# Cerebral Autoregulation during Postural Change in Patients with Cervical Spinal Cord Injury—A Carotid Duplex Ultrasonography Study

**DOI:** 10.3390/diagnostics11081321

**Published:** 2021-07-23

**Authors:** Joo-Hyun Kee, Jun-Hyeong Han, Chang-Won Moon, Kang Hee Cho

**Affiliations:** 1 Department of Rehabilitation Medicine, School of Medicine, Chungnam National University, Daejeon 35015, Korea; awngusla@naver.com (J.-H.K.); jhhan1125@cnuh.co.kr (J.-H.H.); mcw0171@cnuh.co.kr (C.-W.M.); 2 Department of Biomedical Institute, Chungnam National University, Daejeon 35015, Korea

**Keywords:** cervical spinal cord injury, cerebral autoregulation, orthostatic hypotension, cerebral blood flow, carotid duplex ultrasonography

## Abstract

Patients with a spinal cord injury (SCI) frequently experience sudden falls in blood pressure during postural change. Few studies have investigated whether the measurement of blood flow velocity within vessels can reflect brain perfusion during postural change. By performing carotid duplex ultrasonography (CDU), we investigated changes in cerebral blood flow (CBF) during postural changes in patients with a cervical SCI, determined the correlation of CBF change with presyncopal symptoms, and investigated factors affecting cerebral autoregulation. We reviewed the medical records of 100 patients with a cervical SCI who underwent CDU. The differences between the systolic blood pressure, diastolic blood pressure, and CBF volume in the supine posture and after 5 min at 50° tilt were evaluated. Presyncopal symptoms occurred when the blood flow volume of the internal carotid artery decreased by ≥21% after tilt. In the group that had orthostatic hypotension and severe CBF decrease during tilt, the body mass index and physical and functional scores were lower than in other groups, and the proportion of patients with a severe SCI was high. The higher the SCI severity and the lower the functional score, the higher the possibility of cerebral autoregulation failure. CBF should be assessed by conducting CDU in patients with a high-level SCI.

## 1. Introduction

Significant cardiovascular and autonomic dysfunction is a common consequence of high-level spinal cord injuries (SCIs) [1]. Patients with an SCI frequently experience a sudden reduction in blood pressure (BP) upon postural change, which is characterized by dizziness, light-headedness, or even syncope. Orthostatic hypotension (OH) is very difficult to control and severely impairs the quality of life [2]. The symptoms associated with OH significantly interfere with the rehabilitation of patients with an SCI. BP reduction that is diagnostic of OH occurs in 74% of SCI patients, and symptoms of OH (such as light-headedness or dizziness) occur in 59% of individuals with an SCI during physiotherapy [3].

Possible mechanisms underlying OH in SCI patients include changes in sympathetic activity, altered baroreflex function, lack of skeletal muscle pumping activity, cardiovascular deconditioning, and altered salt and water balance [4,5]. However, some people are relatively insensitive to low BP and can maintain consciousness despite low arterial pressure [4]. In these individuals, despite low perfusion pressure, there is a change in cerebral autoregulation (CA) that maintains cerebral blood flow (CBF). Therefore, CA, and not systemic BP, is the predominant factor responsible for the symptoms of OH [6].

Few studies have investigated whether the measurement of blood flow velocity within vessels can reflect brain perfusion during postural change. The blood flow velocity in the middle cerebral artery (MCA) is highly correlated with CBF under conditions of varying mean arterial pressure and cerebrovascular conductance [7,8]. We believe that transcranial Doppler (TCD), which has been mainly used so far, can evaluate cerebral blood flow velocity, but cannot evaluate the cross-sectional area of blood vessels; thus, there is a limit to accurately measuring CBF with TCD. Several studies have shown that the blood flow volume (BFV) of the internal carotid artery (ICA) can be reliably measured, and that it has a close correlation with CBF values [9,10,11]. A previous study used carotid duplex ultrasonography (CDU) as a tool for CBF measurement in patients with brain injury, and it revealed a significant correlation with BFV compared to when TCD was used [12].

Few studies have measured CBF in patients with an SCI and evaluated cerebral hemodynamics during postural changes. In this study, we assessed CBF by using CDU to measure changes in the internal carotid blood flow during postural changes.

The purpose of this study was to investigate, with CDU, the changes in cerebral blood flow volume (CBFV) after postural change, using a tilt table in patients with tetraplegia due to a cervical spinal cord injury (CSCI). We aimed to confirm the correlation of CBFV with symptoms of OH and evaluate the necessity of applying this test in actual clinical practice. In addition, we aimed to determine whether the degree of the SCI was related to changes in CBF when standing, and to elucidate the factors that affect CA.

## 2. Materials and Methods

### 2.1. Study Design

This study was a retrospective review of the medical records of all patients who underwent CDU after a CSCI at the Department of Rehabilitation Medicine, Chungnam National University Hospital, from 1 January 2018 to 31 December 2019.

Patients with complete or incomplete tetraplegia due to a CSCI and who were 18 years of age or older were included in the study. We excluded cases with insufficient medical records or insufficient test results to explain hemodynamic changes, cases with history of fatal cardiovascular disease, and cases of unstable SCIs. Finally, the medical records of 100 patients were reviewed.

### 2.2. Protocol of Carotid Duplex Ultrasonography

**Figure 1:**
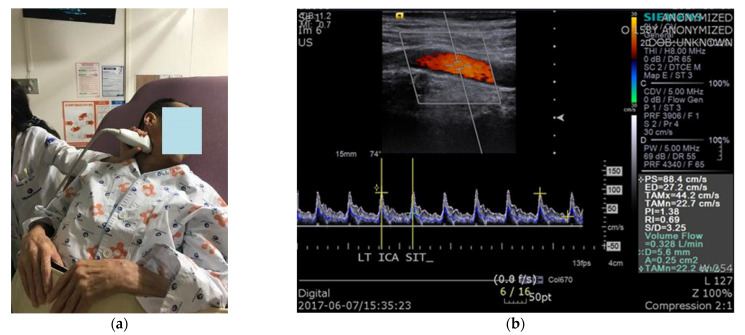
Carotid duplex sonography: (**a**) The position of the patient and measurements of cerebral blood flow using carotid duplex ultrasonography; (**b**) The value of interest in the test was volume flow.

We conducted CDU during head-up tilt in patients with a CSCI. The ICA was studied on both sides, and intravascular flow volumes were calculated using a 9-MHz linear array transducer. BFV measurements were automatically calculated by the built-in software of the ultrasound device (Siemens ACUSON, Siemens Healthcare, Erlangen, Germany). For flow-volume measurements, the head was turned 25°–40° to the contralateral side, and a straight segment of the ICA, at least 2 cm above the carotid bulb, was selected (Figure 1). Measurements were performed on a horizontal segment in the sagittal plane. The arterial diameter was calculated as a vertical line through the lumen between the echogenic intimal layers. The value obtained from the test is volume flow rate, which was automatically calculated from the cross-sectional area of the blood vessel and the time-averaged mean velocity: volume flow rate = area (cm^2^) × time-averaged mean velocity (cm/s). The peak systolic velocity, end diastolic velocity, time-averaged mean velocity, vessel diameter, and vessel area were all measured during the test. All patients were allowed to rest on the examination table for 5 min before the test. CBFV, BP, and heart rate (HR) were measured in the supine position, immediately after the patient was tilted by 50°, and 5 min after the tilt. To increase the reliability of the test, CBF was measured three times in each position, and the mean value was used. In addition, the presence or absence of presyncopal symptoms, such as dizziness, light-headedness, nausea, and blurry vision, in each position, was recorded.

### 2.3. Data Acquisition and Data Analysis

The data obtained during the ICA Duplex ultrasonography were systolic blood pressure (SBP), diastolic blood pressure (DBP), HR, average BFV, and presence of OH symptoms in each position. In order to determine whether the patient experienced OH symptoms when BFV (L/min) was reduced to some extent during the tilt, after obtaining the BFV difference (BFV difference (%) = BFV(supine) − BFV(tilt 50° or 5 min)/BFV(supine) × 100) between the value measured in the supine posture and the value measured after 5 min at 50° of tilt, the point with the highest sensitivity and specificity was obtained by applying the receiver operating characteristic (ROC) curve to the relationship between the BFV difference and the presence of symptoms. It was assumed that there would be symptoms of OH when CBF fell below that point.

**Figure 2:**
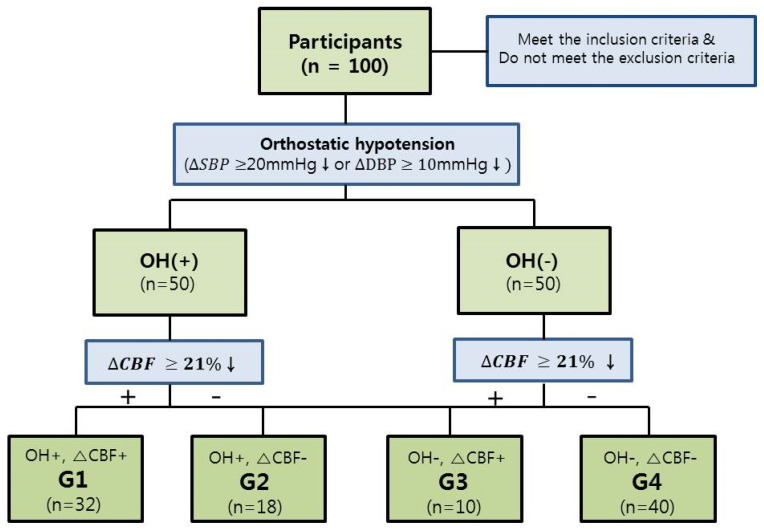
Recruitment of patients and division into groups for data analysis. OH, orthostatic hypotension; CBF, cerebral blood flow; SBP, systolic blood pressure; DBP, diastolic blood pressure.

We calculated the difference between the SBP, DBP, and CBFV values measured in the supine position and the values measured after 5 min at 50° tilt. Based on the presence of OH or ΔCBF, the patients were divided into four groups. The presence of OH was determined according to criteria defined by The American Autonomic Society and the American Academy of Neurology (OH was defined as a decrease in SBF of at least 20 mm Hg or a decrease in DBF of at least 10 mm Hg within three minutes of standing up). If there was a difference in BFV beyond the set point of the presyncopal symptoms mentioned above, it was marked as ΔCBF+; otherwise, it was marked as ΔCBF−. As shown in Figure 2, patients in group 1 (G1) had OH and decreased CBF (OH+, ΔCBF+), those in group 2 (G2) had OH but preserved CBF (OH+, ΔCBF−), those in group 3 (G3) did not have OH but had decreased CBF (OH−, ΔCBF+), and those in group 4 (G4) had neither OH nor decreased CBF (OH−, ΔCBF−).

Data on age, height, body mass index (BMI), duration of injury (DOI), American Spinal Cord Injury Association impairment scale (AIS) grade, sex, neurological level of injury (NLI), and underlying disease (such as diabetes mellitus (DM) and hypertension (HTN)) were collected in each group. To confirm the relationship between functional status and CA, the motor score (MS), sensory score (SS), and Korean spinal cord independence measure (K-SCIM) score were assessed. In addition, the presence or absence of presyncopal symptoms after 5 min at 50° tilt was noted in each group.

### 2.4. Statistical Analysis

The ROC curve was used to determine whether the patient experienced symptoms when the BFV (L/min) decreased to a certain extent at 5 min after 50° tilt. A one-way analysis of variance was used to compare differences in age, height, BMI, DOI, MS (upper extremity, UE), MS (lower extremity, LE), total MS, SS (light touch, LT), SS (pin prick, PP), total SS, and K-SCIM among the four groups. For post-analysis, the Scheffe test was used to determine whether the differences among the groups were significant. The data are reported as mean ± standard error. Crossover analysis and chi-squared test were used to evaluate differences in distributions of AIS grades, sex, NLI, DM, HTN, and the presence of presyncopal symptoms among the four groups. All statistical analyses were performed with IBM Statistical Product and Service Solutions software, version 26.0 (IBM Corporation, Armonk, NY, USA). Statistical significance was set at *p* < 0.05.

## 3. Results

### 3.1. The Relationship between the Decrease in CBFV (L/min) after Tilt and Presence of Presyncopal Symptoms

**Figure 3:**
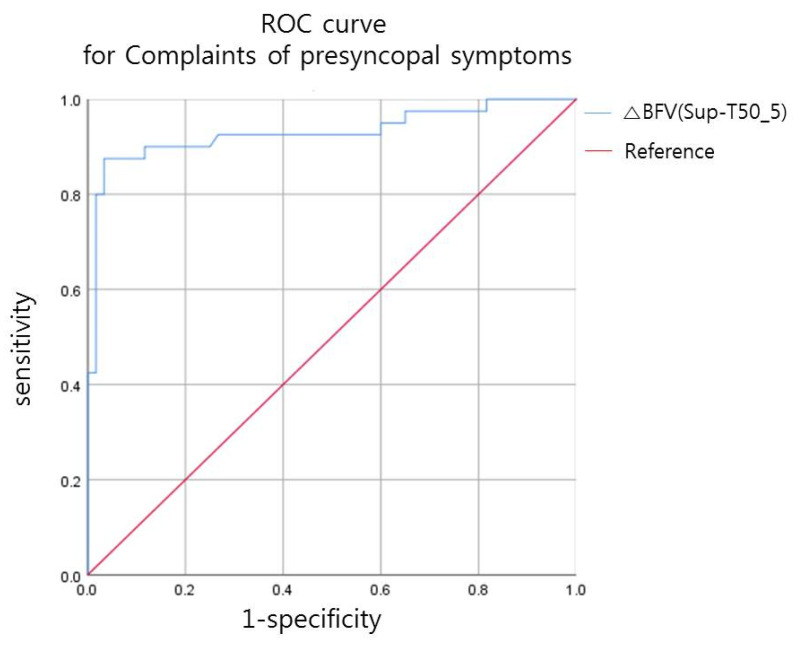
Receiver operating characteristic (ROC) curve for the relationship between decrease in blood flow volume (BFV, L/min) after tilt and the presence of presyncopal symptoms.

Of the 100 patients included in the study, 40 complained of presyncopal symptoms during tilt. The ROC curve of the difference in CBFV after tilt in the presence of presyncopal symptoms is shown in Figure 3, and the data are further presented in Table 1. According to the analysis of the ROC curve, presyncopal symptoms occurred when CBFV decrease was more than 21% after tilt, with a sensitivity of 0.875 and specificity of 0.967 (Table 1).

### 3.2. Demographic Data of Groups and Comparison of Characteristics of Each Group

G1 (OH+, ΔCBF+) had 32 patients, G2 (OH+, ΔCBF−) had 18, G3 (OH−, ΔCBF+) had 10, and G4 (OH−, ΔCBF−) had 40 patients, as shown in Figure 2. There was no statistically significant difference in age, height, and DOI among the groups, but there was a significant difference in BMI among the groups (Table 2). The difference in AIS grades among the groups was also significant (Table 3). In G1, the number of patients with AIS grades A, B, and C was 26 out of 32 (81.25%), which was much higher than that of the other groups. The proportions of the AIS grades in G1 were 92.31% for AIS grade A (12 out of 13 patients), 75.00% for AIS grade B (3 out of 4), 57.89% for AIS grade C (11 out of 19), and 9.38% for AIS grade D (6 out of 64). In G4, 54.69% of patients (35 out of 64) had AIS grade D injury, which was higher than the proportion of AIS grades A, B, and C injuries. Sex, NLI, and presence of underlying disease (DM and HTN) were not significantly different among the groups (Table 3). When comparing the functional scores among the four groups, there were significant differences in the MS, SS, and K-SCIM scores (*p*-value < 0.05, Table 4). In multiple comparisons, the average BMI in G4 was 3.095 kg/m^2^ (*p* = 0.006) higher than that in G1. MS (UE), MS (LE), MS (total), and K-SCIM scores were lower in G1 than in the other groups, and the differences were statistically significant. SS (LT), SS (PP), and SS (total) values in G1 were also significantly lower than those in G4 (*p* < 0.05).

### 3.3. The Incidence of Presyncopal Symptoms in Each Group

**Figure 4:**
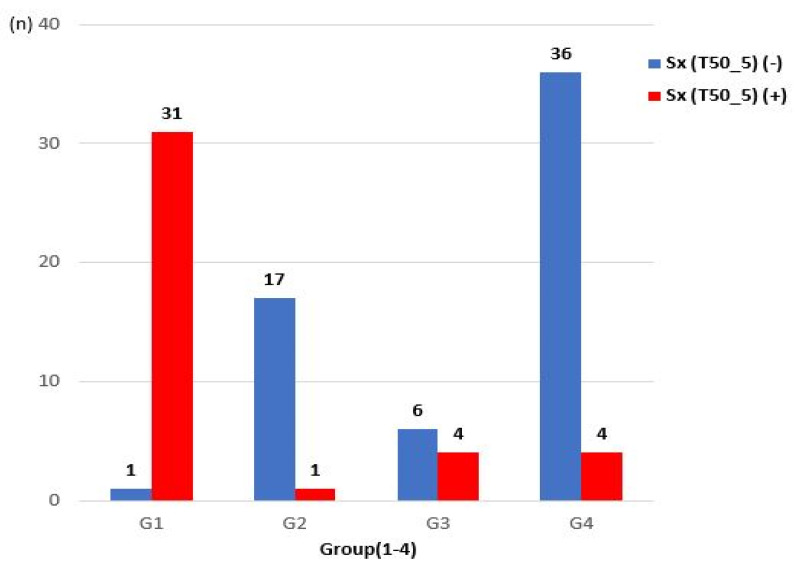
Comparison of the occurrence of presyncopal symptoms among groups.

Presyncopal symptoms were found in approximately 96.88% of patients in G1, 5.56% in G2, 40% in G3, and 10% in G4 (Figure 4). In G2, even if there was a drop in BP during tilt, since CBF was maintained, symptoms rarely occurred.

## 4. Discussion

To our knowledge, this is the first study to use CDU to investigate changes in CBF during postural change in patients with a CSCI. The main findings are as follows:

1. Presyncopal symptoms occurred when the BFV of the ICA decreased by ≥21% after tilt in patients with a CSCI.
2. In G1, i.e., patients who had OH and severe CBF decrease during tilt (because CA did not occur), the BMI was lower than that in G4 (patients who had neither OH nor CBF decrease); physical and functional scores such as MS (UE), MS (LE), MS (total), SS (LT), SS (PP), SS (total), and K-SCIM scores were low; and the proportions of AIS grades A, B, and C were high.
3. In G2, i.e., the group of patients who had no decrease in CBF even though there was OH during tilt (because CA occurred), presyncopal symptoms rarely occurred (5.56%).

The mechanism that underlies OH after an SCI remains unclear. According to some studies, if there is a problem in the pathway from the motor center to the sympathetic nerves due to a high-level SCI, the activation of the sympathetic nervous system through baroreceptors and chemoreceptors fails. Therefore, when external factors that can affect BP, such as postural changes, occur, the mechanism of maintaining normal BP does not work. Therefore, in the early stages of an SCI, OH is a result of decreased sympathetic nerve response, venous vasodilation, abdominal muscle paralysis, and inadequate secretion of hormones [4,13]. However, over time, the distal sympathetic preganglionic neuronal conduction pathways of patients with an SCI adapt, and OH improves through the partial recovery of sympathetic nerve function, increased secretion of vasoconstrictors, and increased susceptibility of blood vessels to these vasoconstrictors [14]. In addition, studies have shown that compensation through the kidneys is an important part of the long-term adaptation mechanism for sympathetic nervous system abnormalities [15]. Another study reported increased sensitivity to arginine vasopressin in patients with an SCI [16]. On the other hand, animal experiments in one study showed that neither the sympathetic nervous system nor arginine vasopressin had any effect on BP fluctuations after an SCI [17].

In 1991, a study using TCD suggested that the difference in CA, which regulates CBF, rather than changes in BP, would be involved in symptomatic improvement in patients with sympathetic nervous system abnormalities due to an SCI [6]. Since then, several studies have investigated CBF control in patients with a high-level SCI using TCD [18,19]. CA involves myogenic, metabolic, and neurogenic control mechanisms, as well as systemic factors [20,21], and while static CA is well preserved in patients with a high-level SCI, dynamic CA is severely altered [22,23]. If CA fails, irreversible neuronal cell death can occur [24]. Therefore, the importance of CA for maintaining CBF during tilt has been recognized. A previous study has also assessed whether CBF increased when midodrine, a drug for treating OH, was administered [25].

In the aforementioned studies of CBF in patients with an SCI, TCD was used to assess the flow velocities, but the cross-sectional areas of blood vessels could not be evaluated; therefore, there is a limit to measuring the exact CBFV with TCD. For this reason, we used CDU to measure blood flow, and we measured ICA BFV as an estimate of CBF. Previous studies have measured ICA BFV to estimate CBF [9,10,11,12], confirming that it shows a significant correlation with CBFV, compared to TCD [12]. To our knowledge, this study is the first to measure changes in CBF in patients with an SCI by performing CDU, and we found that presyncopal symptoms occurred when ICA blood flow was reduced by ≥21% with the patient in a tilted position. It was confirmed once again that CA, which modulates CBF, is important in the presence or absence of OH symptoms. The incidence of OH after an SCI is related to the level of injury, degree of damage, time spent lying in bed, physical function, etc. The more severe the paralysis, the higher the incidence rate of OH [26]. Consistent with this, in our study, the physical and functional scores in the group in which CA failed were significantly lower than those in the group in which CBF was maintained, and the proportion of AIS grades A, B, and C (i.e., the proportion of patients with a relatively severe SCI) was high in the former group. Regarding the correlation with presyncopal symptoms, we believe that CBF was preserved in G2, and the symptom-free rate was, therefore, high (only 5.56% had OH symptoms). In this group, even when OH was confirmed, there was no need to discontinue the rehabilitation treatment with tilt, and it was not necessary to administer drugs such as midodrine. Contrastingly, in G3, there was no decrease in BP during tilt, but CBF had decreased by more than 21% resulting in presyncopal symptoms in 40% of patients, which required the consideration for the administration of an anti-OH drug. Since CBF is often reduced in patients with OH symptoms due to CA failure, it is appropriate to measure CBF as well as BP in clinical practice, and to administer drugs if there is up to 21% decrease in CBF. Additionally, in clinical practice, patients with a high-level SCI often discontinue rehabilitation treatment, such as tilt table application due to reduction in BP. In these patients, if CA is ascertained by measuring CBF and there are no symptoms, treatment can be continued and progress can be observed even if there is a drop in BP. Overall, to confirm orthostatic tolerance, both BP and CBF should be assessed in order to properly implement the treatment for OH.

### Limitations

CDU was used to assess CBF in this study. Previous studies have measured regional CBF according to changes in BP using TCD, and some studies have shown that the ICA/MCA region is more sensitive to orthostatic challenges than the vertebral artery/posterior cerebral artery region [25,27,28]. Further studies are needed to determine the extent to which ICA blood flow can reflect total CBF. In addition, several factors, such as blood viscosity/hematocrit and intracranial pressure, may affect blood flow velocity, but these factors were not controlled in this study. Future studies will need to take these factors into consideration. When performing CDU, it is important to measure the cross-sectional area of the artery at a certain location and time. In this study, CDU was performed by more than one person; thus, there might have been inter-observer differences in the data obtained, depending on the proficiency and skills of the observers. Moreover, this study is a retrospective, non-blinded study; the examiner already knew about the patient’s condition when performing the examination, and this may have introduced some bias. Furthermore, the differences in DOI were not considered. A future study that correlates changes in CBF with DOI in individual patients can help elucidate the cerebral hemodynamics over time.

Patients with an SCI suffer from voiding problems, for which most of them take medications such as α-blockers, which can cause OH. This study did not find out whether patients were taking such medications, and this should be considered in future studies. Another limitation is that only the presence or absence of presyncopal symptoms was noted; their severity, which would be more useful for determining the degree of orthostatic tolerance, was not considered. In order to obtain a more accurate conclusion, future studies should include more patients and have a longer follow-up period. There is no precise guideline on whether to administer an anti-OH drug if the SBP falls but the CBF is preserved. In a future study, the follow-up of changes in CBF and prognosis after treatment of OH will be helpful in establishing guidelines for further treatment.

## 5. Conclusions

This study used CDU to confirm changes in CBF during postural changes in patients with a CSCI. If CBFV decreased by more than 21%, presyncopal symptoms occurred. However, even in the presence of OH, if CBFV was preserved (i.e., if CA occurred), patients were less symptomatic. The higher the SCI severity and the lower the functional score, the higher the possibility of CA failure. It is, therefore, necessary to use CDU to assess CBF in patients with a high-level SCI, in order to ensure proper administration of drugs and smooth rehabilitation, and to ultimately improve the quality of life of patients.

## Figures and Tables

**Table 1:** Decrease in BFV after tilt, resulting in presyncopal symptoms.

	AUC	95% CI	Cut-Off Value (%)	Sensitivity	Specificity
ΔBFV (Sup-T50_5)	0.93	(0.870~0.991)	21.23	0.875	0.967

ΔBFV (Sup-T50_5) (%) = BFV (supine) − BFV (tilt 50°, 5 min)/BFV (supine) × 100. BFV, blood flow volume; AUC, area under the receiver operating characteristic curve; CI, confidence interval.

**Table 2:** The demographic data and characteristics of patients in the four groups.

Characteristic, Mean ± SD	G1 (*n* = 32)	G2 (*n* = 18)	G3 (*n* = 10)	G4 (*n* = 40)	Total (*n* = 100)
Age (years)	58.5 ± 16.97	60.61 ± 14.74	63.00 ± 18.94	57.22 ± 13.50	58.82 ± 15.34
Height (cm)	169.41 ± 7.98	163.63 ± 9.31	164.18 ± 13.50	167.20 ± 8.41	166.96 ± 9.16
BMI (kg/m^2^) *	20.92 ± 3.44	23.38 ± 4.54	23.078 ± 2.60	24.01 ± 3.49	22.82 ± 3.80
DOI	185.17 ± 432.04	145.95 ± 188.82	118.89 ± 142.34	158.29 ± 222.59	160.74 ± 293.85

BMI, body mass index; DOI, duration of injury; G1, group 1; G2, group 2; G3; group 3; G4, group 4; SD, standard deviation; * *p* < 0.05.

**Table 3:** The demographic data and characteristics of patients in the four groups.

		G1 (*n* = 32)	G2 (*n* = 18)	G3 (*n* = 10)	G4 (*n* = 40)	Total (*n* = 100)
Characteristic						
AIS grade (*n*) *	AIS A	12	0	0	1	13
	AIS B	3	0	1	0	4
	AIS C	11	3	1	4	19
	AIS D	6	15	8	35	64
Sex (*n*)	Male	26	13	8	30	77
	Female	6	5	2	10	23
NLI (*n*)	C2	1	0	0	1	2
	C3	4	3	1	2	10
	C4	12	7	5	16	40
	C5	14	8	4	18	44
	C6	0	0	0	2	2
	C8	1	0	0	1	2
DM (*n*)	(−)	31	12	7	38	88
	(+)	1	6	3	2	12
HTN (*n*)	(−)	23	13	7	31	74
	(+)	9	5	3	9	26

AIS, American Spinal Injury Association Impairment Scale; G1, group 1; G2, group 2; G3; group 3; G4, group 4; NLI, neurologic level of injury; DM, diabetes mellitus; HTN, hypertension, * *p* < 0.05.

**Table 4:** Comparison of functional scores among the four groups.

Functional Score (Mean ± SD)	G1 (*n* = 32)	G2 (*n* = 18)	G3 (*n* = 10)	G4 (*n* = 40)	Total	*p*-Value (ANOVA)
MS (UE)	22.25 ± 11.92	34.11 ± 5.95	34.2 ± 7.56	36.33 ± 6.60	31.21 ± 10.59	0.000
MS (LE)	12.03 ± 15.23	33.06 ± 12.71	33.70 ± 18.28	36.93 ± 10.07	27.94 ± 17.12	0.000
MS (total)	34.28 ± 23.11	67.17 ± 15.89	67.90 ± 22.66	73.25 ± 14.92	59.15 ± 25.39	0.000
SS (LT)	56.28 ± 22.49	65.56 ± 13.98	65.30 ± 19.03	70.78 ± 20.92	64.65 ± 20.87	0.032
SS (PP)	52.66 ± 25.03	64.00 ± 13.23	60.80 ± 9.39	73.23 ± 23.11	63.74 ± 22.83	0.002
SS (total)	108.94 ± 46.01	129.56 ± 26.41	126.10 ± 25.80	144.00 ± 43.00	128.39 ± 42.35	0.005
K-SCIM	23.44 ± 21.76	62.44 ± 25.80	57.70 ± 25.74	64.78 ± 25.82	50.42 ± 30.59	0.00000

MS, motor score; UE, upper extremity; LE, lower extremity; SS, sensory score; LT, light touch; PP, pin prick; K-SCIM, Korean spinal cord independence measure; G1, group 1; G2, group 2; G3; group 3; G4, group 4; ANOVA, analysis of variance.

## Data Availability

The data presented in this study are available from the corresponding author upon reasonable request.
